# C3 Glomerulonephritis Associated With Unusual IgG4 Antifactor H in IgG4-related Disease

**DOI:** 10.1016/j.xkme.2025.101019

**Published:** 2025-05-03

**Authors:** Paul Dalmas, Mickael Bobot, Noémie Jourde-Chiche, Julie Bruno, Stéphane Burtey, Laurent Daniel, Carine El-Sissy, Véronique Fremeaux-Bacchi, Antonio Jorquera, Vincent Javaugue, Nicolas Schleinitz, Mikael Ebbo

**Affiliations:** 1 Aix-Marseille Univ, APHM, hôpital de la Timone, Internal Medicine Department, Marseille, France; 2 C2VN, INSERM, INRAE, Aix-Marseille Université, Marseille, France; 3 Department of Nephrology and Kidney Transplantation, hôpital de la Conception, AP-HM, Marseille, France; 4 Laboratoire d’Anatomie Pathologique, hôpital de la Timone, AP-HM, Marseille, France; 5 Department of Immunology, AP-HP, Georges Pompidou European Hospital, Paris, France; 6 Inflammation, Complement and Cancer Team, Cordeliers Research Center, INSERM Unité Mixte de Recherche (UMR) S1138, Paris, France; 7 Department of Pathology and Ultrastructural Pathology, Centre Hospitalier Universitaire, Poitiers, France; 8 Control of the immune response B and lymphoproliferation, CNRS UMR 7276, INSERM UMR 1262, University of Limoges, Centre de référence de l'amylose AL et autres maladies par dépôts d'immunoglobuline monoclonale, Limoges, France; 9 Service de néphrologie et Centre National de référence amylose AL et autres maladies à dépôts d'immunoglobulines monoclonales, Centre Hospitalier Universitaire, Université de Poitiers, Poitiers, France

**Keywords:** Antifactor H, C3 glomerulonephritis, IgG4-related disease

## Abstract

C3 glomerulonephritis (C3GN) is characterized by glomerular aggression mediated by deregulation of the alternative complement pathway. C3GN can be inherited or consequent to acquired autoantibodies, notably against factor H. We report the case of a patient with systemic active IgG4-related disease who presented for acute kidney injury with glomerular proteinuria and hypocomplementemia related to C3GN associated with IgG4-related interstitial nephritis on kidney biopsy. Factor H was low, and antifactor H IgG autoantibody was detected. Detection of other acquired or genetic complement alternative pathway disorders returned negative. After initial failure of oral corticoids and intravenous rituximab, the patient was successfully treated by intravenous cyclophosphamide followed by maintenance therapy with rituximab. Antifactor H autoantibody isotypes were IgG1 and IgG3, mainly as all antifactor H in positive controls but also IgG4, which is unusual. This suggests a link in this case between the oligoclonal expansion of plasma cells in IgG4-related disease and the production of antifactor H antibodies, especially of IgG4 isotype.

C3 glomerulonephritis (C3GN) is characterized by complement dysregulation, leading to predominant glomerular C3 deposits (1). It can result from either inherited or acquired complement defects that drive alternative pathway activation (1). Specifically, acquired deficiency in factor H (FH) due to anti-FH antibodies is responsible for 1% of C3GN cases.^1^

IgG4-related disease (IgG4-RD) is a systemic fibroinflammatory condition. The most affected organs include the salivary glands, lacrimal glands, and the pancreato-hepato-biliary system.^2^ Histology remains the cornerstone of diagnosis, revealing typically storiform fibrosis and polyclonal lymphoplasmacytic infiltrate with IgG4^+^ plasma cells.^2^ IgG4-related kidney disease (IgG4-RKD) accounts for 16% of patients with IgG4-RD^3^ and primarily manifests as tubulointerstitial nephritis, with glomerular involvement being less frequent and consistent with membranous nephropathy.^4^

The purpose of this case report is to describe and discuss the association of IgG4-RD and C3GN related to anti-FH antibodies.

## Case Report

**Figure 1:**
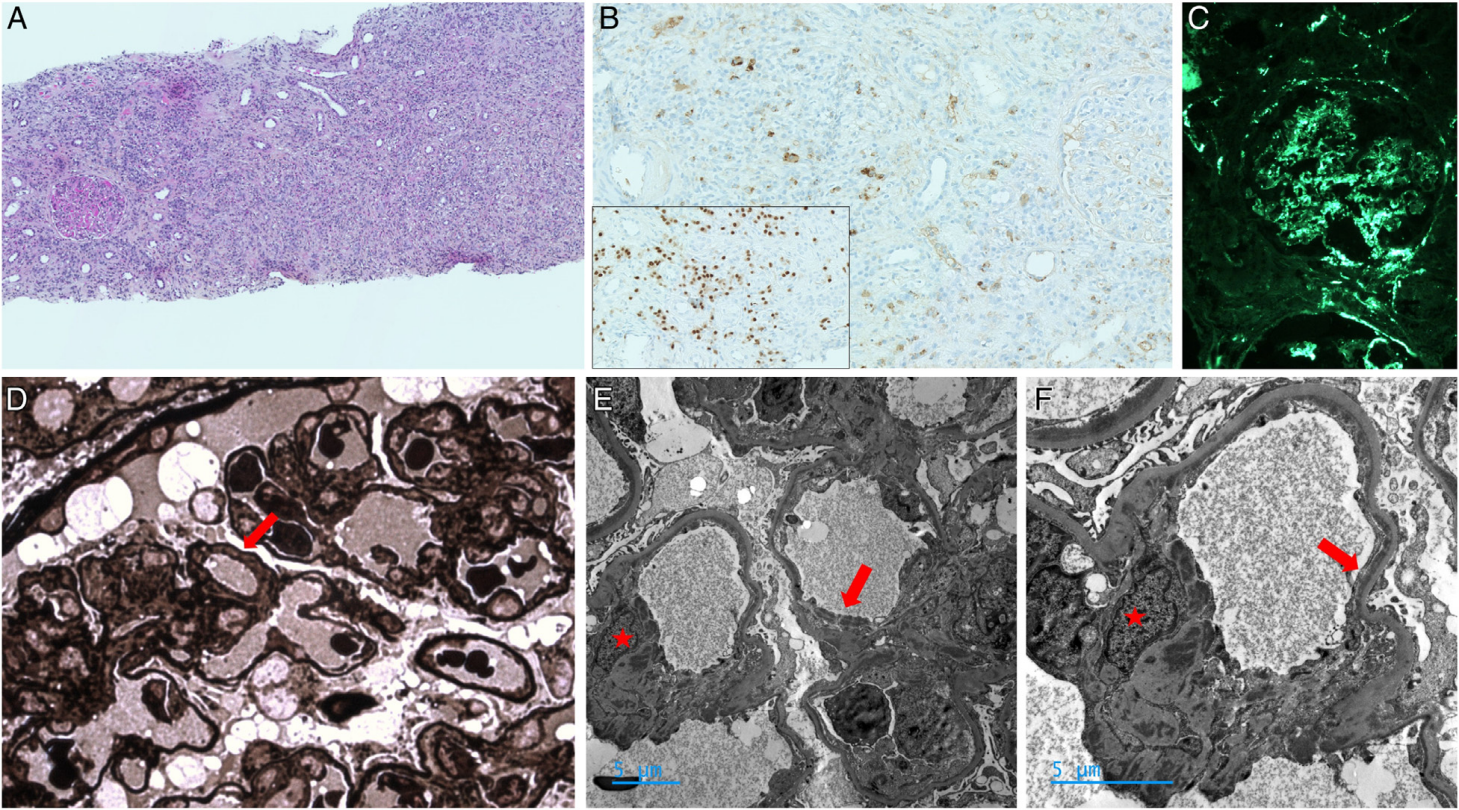
First kidney biopsy. (A) Severe interstitial fibrosis with extensive tubular atrophy and mononuclear inflammation on hematoxylin and eosin staining. Original magnification, ×40. (B) Anti-IgG4 immunohistochemistry showing specific plasma cell staining among total MUM-1 positive plasma cells (insert). Original magnification, ×200. (C) Direct anti-C3-fluorescein isothiocyanate immunofluorescence shows strong labeling within the mesangium and segmental endomembranous spaces and Bowman’s capsule. Original magnification, ×200. (D) Brown deposits are seen within double contours of the capillary wall (arrow) on Jones silver staining. Original magnification, ×400. (E,F) Nodular electron dense mesangial (stars) and interrupted subendothelial (arrows) dense deposits on electron microscopy. Original magnification, ×4,000 (E) and ×7,000 (F).

A 48-year-old patient was admitted due to a deterioration in his overall health. His medical history included class 2 obesity with a body mass index of 35 kg/m^2^, chronic kidney disease (estimated glomerular filtration rate: 55 mL/min/1.73 m^2^, with baseline creatinine at 117 μmol/L) without proteinuria or abnormalities of the urinary sediment, considered as obesity-related chronic kidney disease and IgG4-RD. IgG4-RD was diagnosed a 1.5 years before, with histologically confirmed dacryoadenitis, elevated serum IgG4 (1.4 g/L; normal range: 0.04-0.86 g/L) and decreased C3 (0.73; normal range: 0.81-1.57 g/L) and C4 levels (0.048; normal range: 0.13-0.40 g/L) and CH50 (31%; normal range: 70%-130%). His condition had been effectively managed with oral prednisone for 1 year with an initial dose of 40 mg/d, slowly tapered until reaching 5 mg at month 8 of treatment and pursued at the same posology for 4 months. When the treatment was stopped, the patient had improvement of dacryoadenitis and decrease of serum IgG4 level to 1.18 g/L. However, kidney function still remained impaired but was stable. Six months after prednisone discontinuation, the patient clinically relapsed with left submandibular sialadenitis and bilateral dacryoadenitis. Laboratory tests indicated acute kidney injury with elevated serum creatinine level at 157 μmol/L. C-reactive protein was normal at 2.4 mg/L. Complete blood cell count showed hypereosinophilia at 2.16 × 10^9^/L. Serum protein electrophoresis revealed polyclonal hypergammaglobulinemia (21 g/L; normal range: 8-13.5 g/L) mostly composed of IgG with particularly elevated serum IgG4 at 7.28 g/L, slightly elevated IgG1 at 11.1 g/L (normal range: 3.41-8.94 g/L) and IgG3 at 1.3 g/L (normal range: 0.18-1.06 g/L), normal IgG2 (2.75 g/L; normal range: 1.71-6.32 g/L), and increased serum IgE levels (264 kUI/L; normal range: 1-100 kUI/L). Serum and urinary immunofixation did not detect monoclonal gammopathy. Complement levels were decreased with low C3 (0.05 g/L), C4 (0.017 g/L), and CH50 (<20%). Urinary protein-creatinine ratio was 0.45 g/g creatinine with microscopic hematuria (122/mm^3^) and leukocyturia (19/mm^3^). Tests for anti-neutrophil cytoplasmic, anti-glomerular basement membrane, and anti-phospholipase A2 receptor autoantibodies as well as cryoglobulinemia were negative. Computed tomography scan showed no urinary tract obstruction. A kidney biopsy was performed, revealing dense lymphoplasmacytic infiltrate with storiform fibrosis and an IgG4^+^/IgG^+^ plasma cell ratio of 20% with 10-15 IgG4^+^ plasma cells/high power field. The glomerulus examination revealed endomembranous deposits with a thickening of the glomerular basement membrane with double contours, positive and diffuse immunostaining for C3, leading to the diagnosis of C3GN (Fig 1). Glomerular immunostaining for IgG was mild and negative for IgG4. Serum FH levels were decreased (<12.5%; normal range: 65%-140%) with positive anti-FH antibody (20,000 UA; normal range <100 UA). Screening for C3 nephritic factor was negative. No mutation in the *CFH*, *CFI*, *CD46*, *C3*, *CFB*, or *CFHR5* genes were identified, and no *CFH*/*CFHR1* rearrangement was observed. Treatment included 60 mg of prednisone, 2 infusions of rituximab (1,000 mg on day 1 and day 15), and introduction of angiotensin conversion enzyme inhibitor.

**Figure 2:**
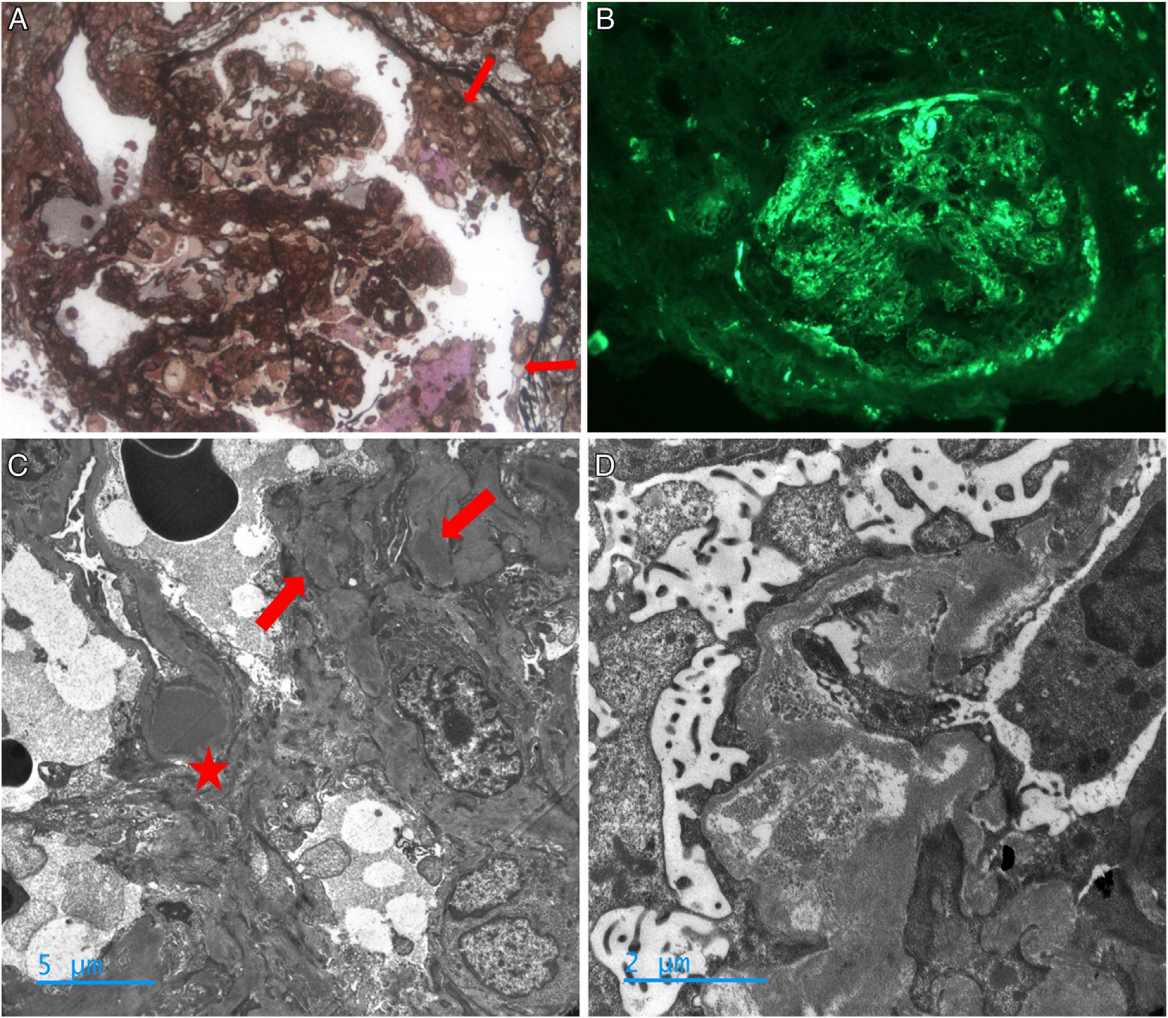
Second kidney biopsy. (A) Segmental cellular crescents with fibrinoid necrosis (arrows) were seen surrounding 2 capillary loops on Jones silver staining. Original magnification, ×400. (B) Direct anti-C3-fluorescein isothiocyanate immunofluorescence again shows staining within the mesangium and segmental endomembranous spaces. Original magnification, ×200. (C) Nodular electron dense humps (star) and subendothelial/paramesiangal dense deposits (arrows) on electron microscopy. Original magnification, ×5,000. (D) Another glomerulus with segmental cellular crescents showing irregular thickening, dissolution of deposits (holes), and sclerosis of glomerular basement membrane on electron microscopy. Original magnification, ×15,000.

**Figure 3:**
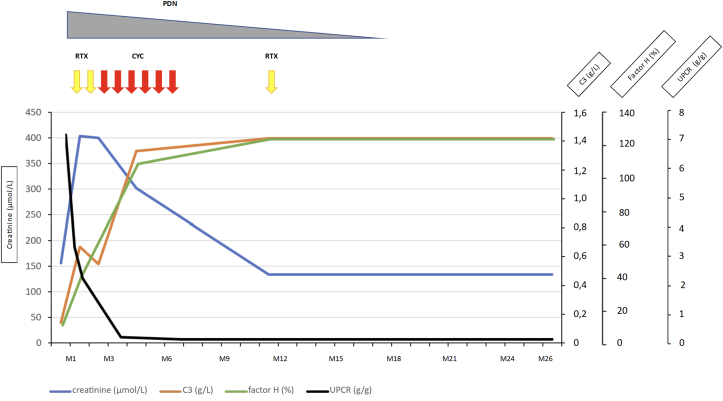
Clinical and biologic evolution. Follow-up time (months) is represented on the x-axis. Laboratory values for creatinine (blue, μmol/L) are shown on the left y-axis and those for factor H (green, %), C3 (orange, g/L), and UPCR (black, g/g) on the right y-axis. The upper part shows treatments with rituximab (yellow arrow, 1g J1 and J15 at induction and 500 mg at maintenance), cyclophosphamide (red arrow, 600 mg/m^2^, intravenous), and prednisone (gray triangle that becomes thinner as the dose is reduced from 60 mg to discontinuation). CYC, cyclophosphamide; PDN, prednisone; RTX, rituximab; UPCR, urinary protein-creatinine ratio.

Two weeks later, the patient developed lower limb edema, macroscopic hematuria, hypertension, acute kidney injury (creatinine: 400 μmol/L), and nephrotic syndrome with glomerular proteinuria (urinary protein-creatinine ratio: 6.1 g/g) and hypoalbuminemia (albumin: 24g/L). Complement level was still decreased with low C3 (0.014 g/L), C4 (0.026 g/L), and CH50 (0%). FH remained low at 40%. A second kidney biopsy revealed extracapillary proliferation with cellular crescents associated with C3GN without interstitial infiltrate (Fig 2). Six courses of intravenous cyclophosphamide 600 mg/m^2^ monthly was administered. At 3 months, proteinuria was undetectable, creatinine decreased to 223 μmol/L, complement level improved (with normalized C3, C4, and CH50 at 27%), and anti-FH was undetectable. Rituximab maintenance at 500 mg was performed 1 year after the first infusion of rituximab and C3, C4, and CH50 were normal. Oral prednisone was discontinued at 18 months. At last follow-up (26 months), the patient was euvolemic, with normal blood pressure and no sign of active IgG4-RD. Creatinine level was 134 μmol/L, estimated glomerular filtration rate was 48 mL/min/1.73m^2^, and proteinuria was negative. Serum IgG4, C3, C4, and CH50 were normal (Fig 3).

### Additional Investigations

To understand the link between IgG4-RD and C3GN, we sought to determine the IgG subclasses of the anti-FH antibodies (Item S1). The patient was positive for anti-FH IgG1, IgG3, and IgG4, whereas all positive controls from atypical hemolytic uremic syndrome were positive only for anti-FH IgG1 and IgG3, but negative for anti-FH IgG4 (Fig S1).

## Discussion

We present a case of C3GN secondary to anti-FH associated with IgG4-RD involving the kidneys, lacrimal glands, and salivary glands. Wallace et al^2^ identified different phenotypes in IgG4-RD, including the pancreato-hepatico-billiary, retroperitoneal and aorta, head and neck-limited, and Mikulicz and systemic clusters.^2^ Our patient can be categorized within this last group. Notably, kidney involvement is more prevalent in this cluster, representing 36% of cases.^2^ IgG4-RKD affects the interstitium in nearly all cases but can also involve the glomeruli in about 16% of cases,^4^ typically presenting with membranous nephropathy.^5^ The double contours of the glomerular basement membrane and strong mesangial and endomembranous positivity for C3 in immunostaining with negativity for IgG4 was suggestive of C3GN in our patient (Fig 1).

To our knowledge, 1 case of C3GN associated with IgG4-RD has been previously reported,^6^ although without details regarding the origin of the C3GN. In the present case, we identified an acquired deficiency in FH due to anti-FH antibodies, resulting in an activation of the alternative complement pathway.^7^ Additionally, anti-FH antibodies, with slightly different immunological properties,^8^ could also be responsible for 4.6%-13% of cases of atypical hemolytic uremic syndrome,^7^ and 1 case of association between atypical hemolytic uremic syndrome secondary to anti-FH and IgG4-RD has been described.^9^

Patients with C3GN and IgG4-RKD both exhibit low complement levels. Interestingly, 45% of IgG4-RKD cases display low levels of C3 and/or C4,^4^ even though IgG4 is a poor activator of the complement system.^10^ Since the initial diagnosis, the patient had shown low levels of C3 and C4, along with a slightly elevated creatinine level, suggesting a potential pre-existing IgG4-RKD in this patient. Finally, after the diagnosis of C3GN, he showed concomitant improvement in complement levels, serum creatinine, urinary protein-creatinine ratio, and FH after treatment with cyclophosphamide and rituximab (Fig 3).

The KDIGO (Kidney Disease: Improving Global Outcomes) 2021 guidelines for the management of C3GN recommend mycophenolate mofetil and corticosteroids as first-line treatment.^11^ However, in our case, we deemed rituximab more appropriate due to the presence of anti-FH autoantibodies, as suggested by Caravaca-Fontán et al,^12^ and considering its efficacy in IgG4-RD.^13^ The initial unfavorable renal outcome of our patient evolving to extracapillary proliferation is rare^14^ (Fig 2), prompting consideration of cyclophosphamide, which has been reported as a successful option in C3GN with crescents^15^ and in proliferative extracapillary glomerulonephritis from other causes.

The patient developed anti-FH related to IgG1, IgG3, and IgG4 isotypes. Associations of IgG4-RD with autoimmune diseases mediated by autoantibodies of IgG4 isotypes have rarely been reported (Table S1). On the other hand, there are many autoantibodies of IgG4 isotype reported without association with IgG4-RD.^16^ IgG4-RD pathophysiology results in fibroinflammatory infiltration with oligoclonal IgG4^+^ plasma cells and subsequent interactions between B and T cells that ultimately drive fibrosis.^17^ IgG4 does not seem to induce tissue injury in IgG4-RD,^17^ and there is no clearly identified specific autoantigen. Conversely, in autoimmune diseases associated with IgG4 autoantibodies, the IgG4 is pathogenic.^16^ In this case, the presence of IgG4 anti-FH antibodies in addition to IgG1 and IgG3 is a unique feature not described previously in diseases mediated by anti-FH,^18^ and this led us to suppose a link between IgG4-RD and C3GN, even if the specific pathogenicity of anti-FH IgG4 cannot be formally proven. Moreover, the fact that C3GN appeared during an IgG4-RD flare in this patient enhances this hypothesis.

In conclusion, we report a case of C3GN associated with unusual IgG4 anti-FH antibodies in a patient with a relapsing IgG4-RD, suggesting a potential pathophysiological link between both diseases. Our case emphasizes the importance of a full analysis of the complement alternative pathway, mainly antibodies against FH, during IgG4-RD associated with glomerular involvement.

## Supplementary Materials

Supplementary File (PDF) Item S1; Figure S1; Table S1.
