# Supplementary material for: C3 Glomerulonephritis Associated With Unusual IgG4 Antifactor H in IgG4-related Disease
Source: Kidney Med. 2025 May 3;7(7):101019. doi: 10.1016/j.xkme.2025.101019 (PMC12173104; doi:10.1016/j.xkme.2025.101019)
Supplement: Supplementary File (PDF) — Item S1; Figure S1; Table S1. [file mmc1.pdf]

Item S1: Material and methods

A 96 well Nunc Maxisorb™ ELISA plate (Thermo Scientific, Waltham, USA) was coated with 50 µl/well of purified factor H (Quidel, San Diego, USA) at 5 µg/ml in PBS and incubated overnight at 4 °C. Plates were washed once with PBS, then blocked with 200 µl PBS/0.1% Tween (PBS-T) per well for 1 h at room temperature. Plates were washed 3 times with PBS-T. Patient samples, 4 negative without anti-FH and 3 positive (from aHUS patients) were tested at serial dilution from 1/50 to 1/6400 in PBS-T and 50 µl applied on plates. Patient’s sample and controls were incubated for exactly 1 h at room temperature followed by 3 washes with PBS-T. Respectively a 1/800, 1/200 and 1/200 dilution in PBS-T of HRP conjugated goat anti-human IgG1 (SouthernBiotech, Birmingham, USA), IgG3 (SouthernBiotech, Birmingham, USA) and IgG4 (SouthernBiotech, Birmingham, USA) (50 µl/well) was then applied for exactly 1 h. Plates were washed 3 times with PBS-T. Enzymatic activity was revealed using the orthophenyldiamine substrate. An ELISA was considered valid when the range between positive and negative control was greater than 0.8 (OD490).

Figure S1: Measurement of serum anti-factor H IgG1, IgG3 and IgG4

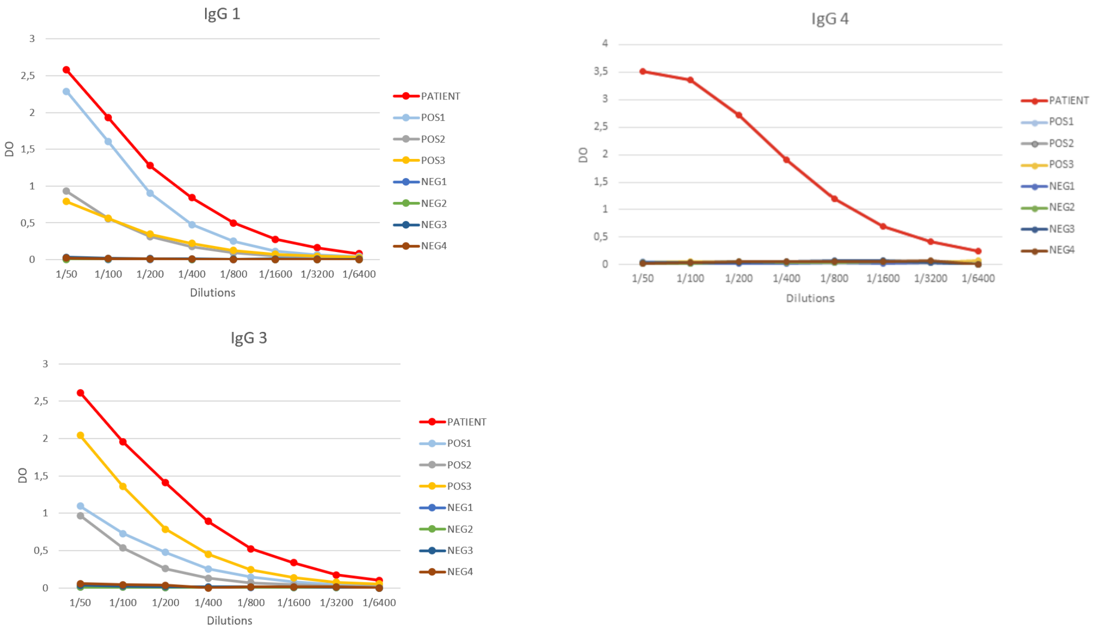

Note: OD: optic density, POS 1,2,3: positive plasma controls (from aHUS patients) with anti-FH antibodies, NEG 1, 2, 3, 4 = negative plasma controls without anti-FH antibodies

Table S1: Reported cases of IgG4-RD with auto-immune diseases associated with autoantibodies of IgG4 isotypes

Report	Age, gender	Auto-immune diseases mediated by IgG4	Autoantibody	IgG4 RD involvement	IgG4 level (g/l)
Sanges et al (1)	75, M	acquired hemophilia	anti-factor VIII	lymph node	11.7
Narazaki et al (2)	64, F	acquired hemophilia	anti-factor VIII	pancreas	1.3
Breville et al (3)	45, F	hemolytic and uremic syndrome	anti-factor H	lymph nodes, salivary glands, lacrimal glands	1.2
Saeki et al (4)	57, M	immune mediated thrombotic thrombocytopenic syndrome	anti-ADAMTS13	lung	2.5
Murase et al (5)	73, M	immune thrombocytopenic purpura	antiplatelets	pancreas	4.7
Ishibuchi et al (6)	70, M	Membranous nephropathy	anti-PLA2R	kidney	2.6

Note: F: female; M: male; IgG4-RD: IgG4 related disease; ADAMTS13: a disintegrin and metalloprotease with thrombospondin type I repeats-13; PLA2R: phospholipase A2 receptor.

Supplementary References

1.Sanges S, Jeanpierre E, Lopez B, et al. Acquired hemophilia A in IgG4-related disease: case report, immunopathogenic study, and review of the literature. *Front Immunol*. 2020;11:558811. Published 2020 Dec 18. doi:10.3389/fimmu.2020.558811.
2.Narazaki T, Haji S, Nakashima Y, et al. Acquired hemophilia A associated with autoimmune pancreatitis with serum IgG4 elevation. *Int J Hematol*. 2018;108(3):335-338. doi:10.1007/s12185-018-2441-3.
3.Breville G, Zamberg I, Sadallah S, Stephan C, Ponte B, Seebach JD. Case report: severe complement-mediated thrombotic microangiopathy in IgG4-related disease secondary to anti-factor H IgG4 autoantibodies. *Front Immunol*. 2021;11:604759. Published 2021 Feb 11. doi:10.3389/fimmu.2020.604759.
4.Saeki T, Ito T, Youkou A, et al. Thrombotic thrombocytopenic purpura in IgG4-related disease with severe deficiency of ADAMTS-13 activity and IgG4 autoantibody against ADAMTS-13. *Arthritis Care Res (Hoboken)*. 2011;63(8):1209-1212. doi:10.1002/acr.20484.
5.Murase K, Matsunaga T, Hayashi T, et al. Successful treatment of autoimmune pancreatitis complicated with autoimmune thrombocytopenia and interstitial pneumonia by prednisolone. *Intern Med*. 2008;47(11):1033-1038. doi:10.2169/internalmedicine.47.0803.
6.Ishibuchi K, Iwakura T, Ema C, et al. A case of M-type phospholipase A2 receptor-associated membranous nephropathy with IgG4-positive cells infiltration in the interstitium. *Clin Med Insights Case Rep*. 2022;15:11795476221078635. Published 2022 Feb 23. doi:10.1177/11795476221078635.
